# Barriers to and solutions for addressing insufficient professional interpreter use in primary healthcare

**DOI:** 10.1186/s12913-019-4628-6

**Published:** 2019-10-25

**Authors:** Fabienne N. Jaeger, Nicole Pellaud, Bénédicte Laville, Pierre Klauser

**Affiliations:** 1 Kollegium für Hausarztmedizin, Rue de l’Hôpital 15, CH-1701 Berne, Fribourg Switzerland; 2 Swiss Tropical and Public Health Institut, Socinstrasse 57, CH-4002 Basel, Switzerland; 3 University of Basel, CH-4003 Basel, Switzerland; 4 Swiss Society of Paediatrics, Rue de l’Hôpital 15, CH-1700 Fribourg, Switzerland

**Keywords:** Language barrier, Interpreter, Primary care, Paediatric, Family doctors, Migrant, Immigrant, Access, Financing, Health services organization

## Abstract

**Background:**

The aim of this nationwide study was to investigate barriers to adequate professional interpreter use and to describe existing initiatives and identify key factors for successful interpreter policies in primary care, using Switzerland as a case study.

**Methods:**

Adult and paediatric primary care providers were invited to participate in an online cross-sectional questionnaire-based study. All accredited regional interpreter agencies were contacted first by email and, in the absence of a reply, by mail and then by phone. Local as well as the national health authorities were asked about existing policies.

**Results:**

599 primary care physicians participated. Among other reasons, physicians identified cumbersome organization (58.7%), absent financial coverage (53.7%) and lack of knowledge on how to arrange interpreter interventions (44%) as main barriers. The odds of organising professional interpreters were 6.6-times higher with full financial coverage. Some agencies confirmed difficulties providing professional interpreters for certain languages at a timely manner. Degrees of coverage of professional interpreter costs (full coverage to none) and organization varied between regions resulting in different levels of unmet needs.

**Conclusions:**

Professional interpreter use can be improved through the following points: increase awareness and knowledge of primary care providers on interpreter use and organization, ensure financial coverage, as well as address organizational aspects. Examples of successful interventions exist.

## Background

With societies becoming more diverse, addressing language barriers in patient care has become a relevant topic [1–6]: In Switzerland, a country with 24.9% of its population being international permanent residents [7], for example, the language barrier is perceived as one of the most relevant challenges by primary care physicians when caring for migrants [8].

Medical consultations may require a high level of language proficiency. Without appropriate communication, taking a patient history, determining a diagnosis and ensuring a well-observed treatment plan is made difficult, potentially hampering the quality of care provided. International research demonstrates reduced rates of unnecessary exams and hospitalisations and costs [1, 4], a reduction of adverse events [1] and hospitalisation durations [9] as well as an increased up-take of preventive measures [2] and patient and provider satisfaction [10] when professional, thus qualified, interpreters are used to overcome language barriers. Primary care physicians in Switzerland, for example, report to relinquish giving health promotion advice or explanations concerning disease and treatments, to order extra exams, referrals to emergency wards and hospitalisations, to feel unable to provide good quality care and even witness complications due to not properly addressed language barriers [11]. Approximately 1/3 believe they could have saved costs using a professional interpreter [11]. These issues are likely to be relevant in other host countries without general access to free-of-charge professional interpreters for primary health care, too, such as, for example, Germany or Croatia [12].

Although not necessarily required for all consultations with a language barrier, professional interpreters may further help clarify intercultural uncertainties [13], which is relevant as intercultural challenges are reported by a majority of primary healthcare providers [11].

While public hospitals may have funds for professional interpreters or may be able to rely on a pool of bilingual staff [14, 15], organising professional interpreters may be more challenging for primary care paediatricians and family doctors. We therefore aim to assess factors relevant to professional interpreter use in primary health care. With previous research from Switzerland demonstrating high unmet needs [11] and the decentralised organisation of the country making it ideal for studying a multitude of policies, we use it as a case study.

Primary healthcare in Switzerland is organised on a private basis with most of the approximately 8000 primary healthcare physicians [16] – family doctors (FD) and primary care paediatricians (PCP) - working in small private or group practices. Mandatory health insurance covers medical but not interpreter costs [17]. The country has 26 regions, called cantons, with each having independent health authorities and administrations. While the overall health system is the same, monitored by the federal government, and the level of care similar, the regional authorities determine what kind of additional programs, such as interpreter services, they want to organise and fund. The number of asylum seekers allocated to cantons is in proportion to the cantons’ resident population. This allows for comparing different policies on a small geographical area with similar parameters.

Currently, only 1/3 of primary care physicians in Switzerland who care for patients despite language barriers benefit from professional interpreters at least once a year [11]. The majority (87.8%) though would appreciate professional interpreter services at least once a year, more than half even at least once a month, and 10% at least once a week [11]. Only a minority of primary care physicians organises professional interpreters themselves [11] despite a clearly expressed need and potential negative consequences in the absence of professional interpreters [8].

The aim of this study is therefore to gain nationally representative insights on barriers to professional interpreter use, to describe existing interpreter policies and to try to identify key elements that are essential for successful interpreter policies in primary care.

## Methods

### Study approach

In order to gain as complete a picture as possible of the current situation and a better understanding of existing barriers and potential solutions to adequate interpreter use, and to be able to triangulate answers, the study had three arms: primary care providers, interpreter agencies and authorities were interviewed aiming for best possible national coverage.

### Primary care providers

The cross-sectional study among primary care providers was part of a larger attempt to investigate professional interpreter use in the non-hospital based general adult and paediatric primary care setting in Switzerland (for more details see [11]). An online questionnaire was developed, piloted and made available in the three main national languages. It focused on (i) the frequency of consultations with a relevant language barrier and the migration population affected by language barriers; (ii) means used to overcome the langue barrier; (iii) attitudes towards, knowledge regarding and actual and intended use of professional interpreters; (iv) unmet professional interpreter needs, (v) main barriers to adequate use of professional interpreters and (vi) desired forms of interpreting (see Additional file 1). In February and March 2017, links to the online questionnaire were sent via email to all members of the Swiss Society of Paediatrics registered as primary care paediatricians (1020 PCP) and all members of Haus- und Kinderärzte Schweiz, the association for political issues of Swiss PCP and FD (Haus- und Kinderärzte Schweiz: 4358 FD; 500 PCP). Both organizations have nationwide coverage. Paediatricians known to be double members received a first email by Haus- und Kinderärzte Schweiz with the reminder email being sent by the Swiss Society of Paediatrics. Participation was anonymous.

Data was analysed descriptively using Stata IC 14. Furthermore Chi-square and, where applicable, logistic regression was applied. Although not the aim of the study, we present results with substantial differences between both participating groups separately.

### Interpreter agencies

All 18 regional interpreter agencies accredited by INTERPRET (national association training and accrediting professional interpreters) and the national telephone interpreter service were contacted by email (November 2016). As return rates were poor, we then sent letters (January 2017) and followed up with phone calls during the first half of 2017 and, in the case of the national telephone interpreter service, by email. Agencies were asked about any knowledge on policies to subsidise professional interpreters for medical consultations in their zone of activity; frequency of interpreters provided for ambulatory medical encounters; services (e.g., languages, lead time needed) provided and interpreter fees charged (see Additional file 2).

### Political authorities

In order to gain insight into currently available services and funding mechanisms, the federal government and all 26 regional (cantonal) authorities were contacted by email, with the exception of Geneva, that had to be contacted by phone. Follow-up phone calls and emails were often helpful to specify information provided by authorities. We requested information on financing mechanisms and projects aimed at improving professional interpreter use by primary care physicians in their jurisdiction The federal government was reached in September and November 2016 while data collection from cantons stretched out over the first half of 2017, because for some cantons multiple emails and follow-up phone calls were necessary.

## Results

A total of 599 clinically active primary care physicians filled in a questionnaire on language barrier and professional interpreter use corresponding to a response rate of 11.6% (PCP: 25.2%; FD: 8.1%). 351 (58.6%) work as FD and 247 as PCP. The physicians facing language barriers when caring for allophone patients were the focus of this investigation (90.8%, total *n* = 538). Language barriers were defined as the inability to directly communicate well with the patient or in the paediatric setting, with the caregiver. This included 238 paediatricians and 299 FD. Furthermore, a participant working in non-hospital primary care who had failed to state if working as a family doctor or a paediatrician was included in the overall analysis. 246 of the participants were females (52.1%). Excellent coverage of the different regions of Switzerland was obtained with the exception of one small canton (16′000 inhabitants) not represented at all and the Italian part of Switzerland being under-represented [8]. The distribution of work places of paediatricians facing language barriers was similar to the overall study population with 46.0% in cities, 29.1% in urban outskirts and the rest in the countryside (24.9%).

Among the 492 participants, who replied if or not they had already requested any form of professional interpreter services for their private practice, 274 (55.02%) state never having done so. This finding was significantly more frequent (crude OR 1.98, *p* < 0.001; 95%CI: 1.38–2.84) for FD (62.2%; 176/283) than paediatricians (45.3%, 97/214) before adjusting for known financial coverage. Frequency of consultations with a language barrier influenced the likelihood of organising professional interpreter services, with those caring for patients with a language barrier at least weekly being more likely to have organised interpreter services at least once in the past (see Table 1). The participants’ work location and sex had no influence.

**Table 1:** Factors examined with regards to History of having organised Professional Interpreters

Explanatory variables	Organised Interpreters			Adj. OR	*p*-value
	No	Yes	Total		
	N (%)	N (%)	N (%)		
	274 (55.0)	224 (45.0)	498		
Type of primary care provider					
Family doctor	176 (62.2)	107 (37.8)	283 (56.9)	1	
Paediatrician	97 (45.3)	117 (54.7)	214 (43.1)	1.41 (0.84–2.36)	0.19
Frequency of allophone consultations					
≥ 1x/year < 1 mo.	95 (69.9)	41 (30.2)	136 (27.3)	1	
≥ 1x/mo. < 1/week	101 (53.4)	88 (46.6)	189 (37.6)	**2.12** (1.14–3.97)	**0.02**
≥ 1x/week	78 (45.1)	95 (54.9)	173 (34.7)	**2.99** (1.60–5.60)	**0.001**
Sex					
Male	130 (57.5)	96 (42.5)	226 (47.9)	1	
Female	134 (54.5)	112 (45.5)	246 (52.1)	1.04 (0.63–1.72)	0.87
Location					
City	117 (53.2)	103 (46.8)	200 (46.0)	1	
Peri-urban	83 (59.7)	56 (40.3)	139 (29.1)	0.59 (0.32–1.06)	0.08
Rural	68 (57.1)	51 (42.9)	119 (24.9)	1.04 (0.57–1.90)	0.89
Source of Finance known					
No	183 (64.0)	130 (36.0)	286 (81.0)	1	
Yes	12 (17.9)	55 (82.1)	67 (18.98)	**6.62** (3.23–13.58)	**< 0.001**

### Barriers to organising professional interpreters

To assess barriers to appropriate interpreter use, a dual approach was used in order to i) identify the most relevant barriers that need to be addressed as a priority, and in order to ii) also identify further barriers that should ideally be addressed for optimal interpreter policy implementation. Participants were therefore, on the one hand, requested to choose the most relevant reason for not organising professional interpreters and, on the other hand, asked to tick all reasons that apply to them from a list.

30.6% identified lack of knowledge on how to organise interventions, 26.9% organizational aspects, and 25.3% insufficient financial coverage as the main reason. 17.1% stated, managing somehow without interpreter as the main reason. There were no differences in these findings between FD and PCP.

**Fig. 1:**
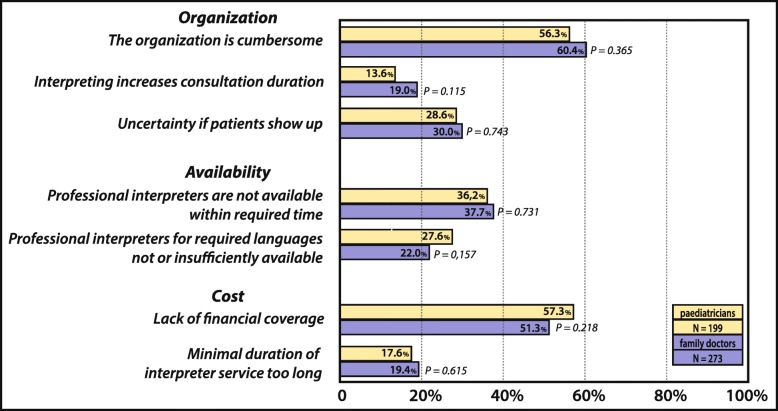
Barriers to Professional Interpreter Use - Part 1: Organisation, Availability and Cost

**Fig. 2:**
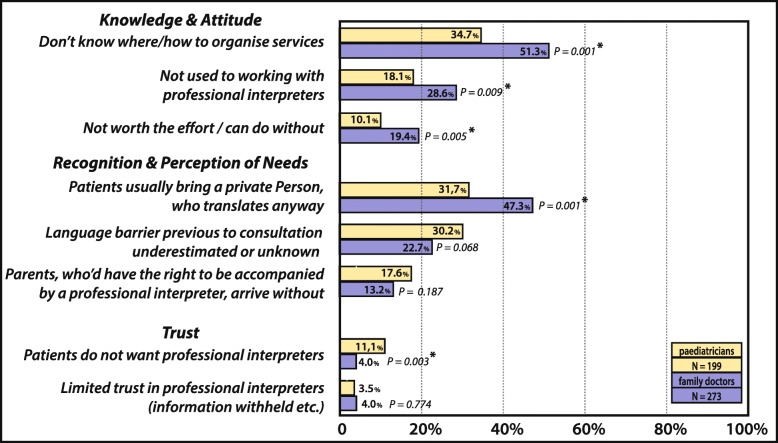
Barriers to Professional Interpreter Use – Part 2: Knowledge & Attitude, Recognition & Perception of Need, Trust

When participants who actually face language barriers were asked to identify all relevant factors for not making use of interpreters more often (see Fig. 1 and Fig. 2), cumbersome organization (58.7%, 277/472) and lack of financial coverage (53.7%,) dominated – both relevant to more than half of all participants. The lack of knowledge on how to organise interventions (44%), was significantly more frequently stated by FD than PCP (*p* < 0.001). FD also tended to more frequently consider efforts not worthwhile than PCP (total 15.4%, FD 19.3%, PCP 10%, *p* = 0.005), to think patients would bring a family-member or acquaintance to do the translation (40.7%, FD: 47.3%, PCP: 31.7%, *p* = 0.001), and to state not being used to working with professional interpreters as a reason (24.2%, FD: 28.6%, PCP: 18.1%, *p* = 0.009). Only the rejection of professional interpreters by patients and families, is more frequently stated by paediatricians (PCP 10% vs. FD: 4%, *p* = 0.003). For all other factors no difference between the two professional groups were noted. Interpreter service constraints such as the lack of availability of professional interpreters within a reasonable time (37.1%) and the lack of availability of interpreters proficient in certain languages (24.5%) were also common, whereas the lack of trust in the quality of translations provided by professional interpreters was only rarely mentioned (3.8%).

In free-text comments, participants repeatedly complained about the unmet high costs, making interpreter services unattractive (e.g., phone interpreter fees: 3 Swiss Francs / min, minimum 30 Swiss Francs; http://0842-442-442.ch/kosten.html). Participants felt, it was not fair that physicians should cover costs related to a potential language barrier with allophone patients as the existence of such a barrier is not the physician’s fault. Repeatedly it was suggested, that health insurances – mandatory for all residents and asylum seekers in Switzerland – should cover interpreter-related costs.

### Presence of financial coverage instruments and interpreter use

**Fig. 3:**
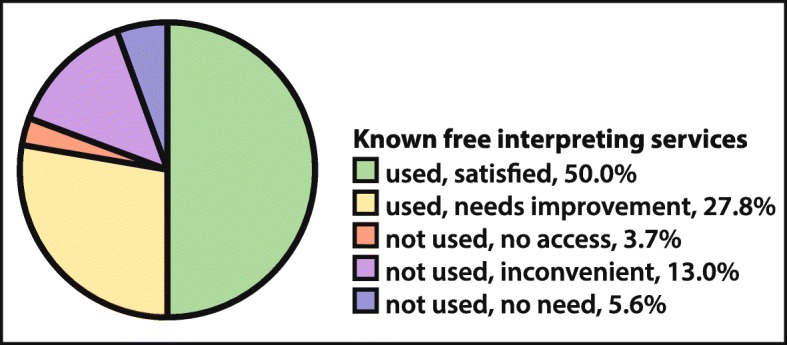
Use of and Satisfaction with Interpreter Programs with Cost Coverage among Physicians aware of such Programs

As financial aspects are among the main reasons not to organise interpreters, we asked participants caring for patients with language barriers whether they had knowledge of any financing resources for interpreter-use in private primary care practices: only 19.0% (67/353) of participants facing language barriers confirmed knowing about them. 4/5 of these knowledgeable participants (82.1%) confirmed having already used the offer – this independent of the medical specialisation. For details on satisfaction with the offer see Fig. 3. Overall, knowledge of available financing instruments increased the likelihood of having organised interpreter services (crude OR: 8.1, 95% CI 4.17–15.91, *p* < 0.001) massively (see also Table 1).

In free-text comments, respondents also state trying to consult together with a parenting counsellor, who can benefit from cantonal interpreting funds, or appeal to social services in case of the suspicion of child abuse or to non-governmental organizations (NGOs) caring for asylum seekers and refugees in order to gain access to an interpreter when other finance structures are unavailable.

### What type of interpreter service is preferred?

**Fig. 4:**
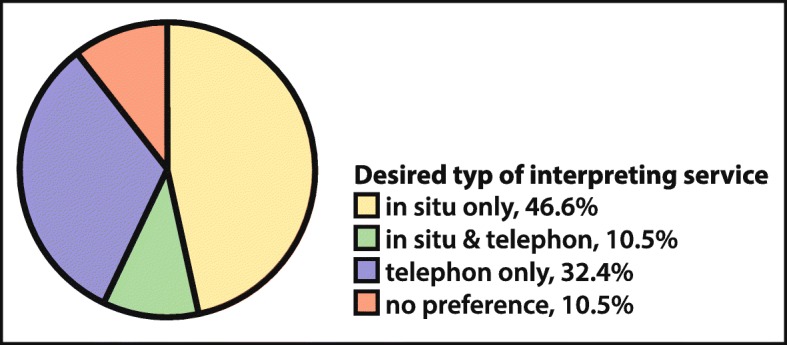
Desired Types of Interpreting

Primary care physicians currently organising professional interpreter services predominantly rely on on-site interpreters (79.0%; 177/224) only, phone-only (4.9%), or a mixture (16.0%). Two thirds of the 476 participants, who face language barriers during their consultations at least once a year, would use free-of-charge interpreter services if made available (68.5%), and only 27 (5.7%) stated they would not, with the remaining (25.8%) undecided or stating they would potentially use it. Figure 4 shows the types of interpreter services that would be preferred, indicating a certain preference for on-site interpreters. Differences between FD and PCP were not significant (*p* = 0.18) put a tendency for PCP preferring a mixture (38.4% vs. 27.5%) of phone and on-site interpreters was noted.

### Available services and experiences with regional efforts to promote interpreter use

Of the 18 regional interpreter agencies and the national telephone interpreter service, only five institutions provided information – all others were not available for information, often due to shortages in personal, the organization not anticipating such requests or restructuring.

Depending on the institution, a half to one-day notice for emergency professional interpreter interventions and 3–5 days for regular interventions are recommended but interventions with a 30-min notice have been reported by bigger interpreter agencies. Still, a smaller agency stated preferring up to 1–2 weeks notice, as their interpreters only translate next to their main activities. A need for training more professional interpreters in Tigrinya, Dari, Farsi and to a lesser extend Arabic is stated. Interpreters for primary care physicians are rarely organised and if so, usually ordered by the authorities. Among the responding institutions only the one in the Italian part of Switzerland (Ticino) seems to organise interventions for FD more frequently (65–75/month) with local authorities paying for interventions regarding asylum seekers.

20 cantons made information available. When not stated otherwise, mentioned cantons are German speaking. Only one administrative region (Grisons, a vast mountainous region in Eastern Switzerland, where next to German also Romansh and Italian are recognised as official languages) reported universal coverage for interpreter use in primary healthcare practices free of charge to physicians and patients. Administrative efforts are kept to a minimum for healthcare providers – simply ordering the interpreter service and signing the professional interpreters’ presence form is sufficient. Despite a slow uptake at first, the program [18], initially financed and set up by the cantonal integration bureau in 2015, is becoming more popular. Still, only 57.1% of participants from that region knew of the offer.

Another small canton (Schaffhausen) had a similar program, though unknown to interviewed health authorities. Several other regional authorities (e.g., Zug; St. Gall) have also tried to promote professional interpreter use, though usually excluding primary care physicians. A reason given for only including e.g., public educational facilities, cantonal out-patient psychiatry and parenting advice services was that primary care physicians work on a private bases. One canton (Basel-Land) had piloted a similar project to that of Grisons granting a 50% discount for PCP when ordering professional interpreters. However, uptake was so slow, that the project was abandoned. Overall, projects usually emerged from the integration offices, experienced a rather slow but then steady uptake and a certain withdrawal of users once finances for the interpreter-projects were cut.

Understanding is also promoted via brochures (e.g., Basel-city; Thurgau) and the co-financing of interpreter services (e.g., Grison, Thurgau, St Gall, both Appenzells, Lucerne) thus helping to reduce interpreter fees. Reaching the medical corps was considered a challenge.

Some cantons pay special attention to asylum seekers and, to a lesser degree, refugees. The degree to which their interpreting needs are met varies greatly depending on the canton and the arrangement the regional authorities have with institutions in charge of forced migrants (NGOs, private, cantonal authorities) regarding interpreter coverage, and the extent to which these are enforced. Initially received by federal reception and procedure centres, asylum seekers are later dispatched to the cantons where they usually first live in asylum centres before moving to apartments. The lack of professional interpreters in federal centres for medical encounters is supposed to be addressed by 2018 (Federal Bureau of Public Health, April 2017). In some regions, assistance is scarce and the use of fellow asylum seekers for interpreting is encouraged; in others, interpreters are available for medical encounters at the asylum-shelter or the asylum-shelter assigned physician’s private practice but they are no longer available once asylum seekers live in their own apartments; other regions provide general coverage of interpreter costs when seeking care from a primary care physician.

The latter is rare: a larger French-speaking administrative area (canton of Vaud) including city and rural parts, allocates paediatric asylum seekers to PCP close to their assigned place of living and adult ones are first followed-up by specialised nurses and later usually addressed to FD in their vicinity. The providers are part of a network of registered primary care providers (Réseaux santé migration – www.resami.ch). They can order professional interpreters entirely financed by the asylum authorities (www.evam.ch). Since June 2016, primary care physicians and psychiatrists can request funding for interpreter services for refugees with a language level up to A2 (basic communication), granted for a period of six months at the time, thus comparing to the duration of language courses. Hoping to increase efficiency and access while reducing costs, this canton is also setting up an online platform to allow more rapid and easier organization of geographically close professional interpreters while also including professional interpreter providers currently not yet accounted for. The Italian-speaking canton of Ticino also covers interpreter costs in primary healthcare and hospital encounters for asylum seekers and refugees who receive social assistance. In the past, Ticino has additionally granted limited funds to cover costs for other migrants requiring interpreters.

A French-speaking small canton in Western Switzerland (Geneva), e.g., tries to allocate adult asylum seekers to language congruent primary care physicians. For asylum seekers with medically more complicated conditions, primary care is provided at the University Hospital, where interpreters are made available. For children, parents may choose a paediatrician, usually sharing their language, or receive primary care paediatric follow-ups at a specialised clinic providing professional interpreters at the University Hospital, which is reachable from throughout the whole city-canton within reasonable time.

Although the cross-sectional study was not designed to detect differences in unmet interpreter needs between cantons, substantially lower unmet interpreter needs for asylum seekers were reported by physicians of the two mentioned French-speaking administrative areas in Western Switzerland (Geneva 7%, Vaud 14%) compared to the national average (51.3%). For some other cantons unmet interpreter needs for asylum seekers as identified by participants reached up to 81.3% (differences among cantons for asylum seekers: *p* < 0.001). Other cantonal interpreter policies included arrangements with local agencies responsible for housing asylum seekers, obliging them to organise interpreters for asylum seekers visiting primary care physicians whenever needed, as long as the consultation was organised via the institution’s nurse (Fribourg, German and French-speaking), or a coverage of interpreter fees limited to the first year (Neuchâtel, French-speaking), a time limit that was much regretted by the participants. Unmet interpreter needs when caring for asylum seekers were identified by 31% of the responding physicians in Fribourg, 40% in Neuchâtel and 50% in Ticino.

Efforts to meet interpreter needs for asylum seekers do not address unmet needs of other groups: Such needs were identified by physician in all cantons without differences reaching significance in this sample. Percentages of physicians identifying unmet needs in non-asylum seeking new-arrivals reached from 12.5% (Zug) up to 61.6 and 58.1% for cantons such as Geneva and Vaud (Swiss mean 38.3% [8]), and percentages identifying such needs for long term foreign residents not having achieved sufficient language proficiency for complex medical encounters reaching from 16.7% (Ticino, Thurgau) to 63% (Fribourg, Swiss mean 33.7% [8]).

Some regional authorities had little overview on available programs and policies regarding migrants and interpreter services. Limited financial resources, uncertainty on who should pay (health versus asylum authorities) and the notion, that generous interpreter services would keep patients from learning the language were mentioned as reasons for a lack of interpreter programs. The federal government supported the national phone interpreter service and considers establishing a nation-wide platform for organization and potentially a video-interpreting trial.

## Discussion

This study clearly demonstrates that various factors influence use of interpreters by primary care physicians starting from perceived need and insufficient knowledge on where to obtain services, to service constraints, as demonstrated by some languages not being available in a timely manner, organizational aspects and funding. It also describes various policies – with lessons learned when implementing them and great differences in the levels of success. Results confirm physicians’ observations that timely presence of professional interpreters is not always available in all languages and that – with exceptions – primary care physicians rarely benefit from their services.

### Financing

Costs linked to interpreter fees, minimal-duration-fees, and work-time spend on organization make interpreter use unattractive for primary care physicians. Financial barriers remain among the main hurdles for adequate interpreter use [8] that need to be addressed primarily. Efforts to provide professional interpreter services free of charge have a great impact: study participants who are aware of financial coverage were more than six times more likely to have organised professional interpreters and full coverage of interpreter costs in two regions have substantially reduced unmet needs for professional interpreters as identified by primary care physicians. A mere reduction but not full elimination of costs though has not proven sufficient as demonstrated by a trial which granted a 50% cost-reduction for primary care paediatricians with insufficient up-take.

With potential cost savings obtained through professional interpreter use mainly concerning the healthcare sector, it seems appropriate, that not only asylum-authorities but also the local and national health departments should contribute to covering interpreter costs. Having the mandatory health insurance pay for interpreter costs is difficult under the current legal framework in Switzerland [17].

### Additional factors

Experiences from Australia clearly demonstrate that providing interpreter services free of charge to general practitioners in an attempt to reduce discrimination and potential litigations and to improve quality of care is not sufficient to ensure their use [19, 20]. Various co-factors that also need addressing have been identified by our study and internationally [19, 20]:

*Timely availability of requested languages* needs to be ensured. The lack hereof has been identified as a barrier by physicians not only in this study but also in other settings [19]. The survey among interpreter agencies confirmed these points and the need to increase training of new professional interpreters and improve organizational aspects.

*Type of provided services needs to match expressed needs*. While most participants favour on-site interpreters or a mixture – on-site is sometimes perceived as more agreeable and easy [8], phone interpreters hold the advantage of rapid availability for short emergency consultations. Video interpreting, though requiring more equipment, may add a more personal touch with the potential of including gestures and non-verbal clues [19].

*Organization needs to be as simple as possible*. Online platforms for easy booking seem promising to reduce organizational hurdles. Keeping administrative efforts of doctors to a minimum had been mentioned being essential (e.g., Grisons).

*Awareness of the benefits of and knowledge on professional interpreter use* should be increased. Knowledge on how and when to use professional interpreters influence their use [19]. Increased use of professional interpreters has been demonstrated after training on how to work with them [21]. It is likely to improve uptake in the primary healthcare non-hospital setting. Not being used to interpreters and not knowing how to organise them have been frequently mentioned in our study same as an underestimation of interpreter needs. Awareness on the benefits of using professional interpreters, including their ability to enhance cultural understanding, should be raised.

*Raising awareness of available services and means of access* [3, 19] is imperative, especially once new, more attractive resources are available, as the lack of such knowledge was among the main barriers identified. Only 57% of participants of the canton (Grisons) that provided universal coverage of interpreter fees were aware of the service. For interventions to be successful, primary care physicians need to be repeatedly informed, preferably using multiple information channels such as professional organizations and associations, conferences and accredited trainings. Front line staff, such as receptionists giving out appointments, also needs to be trained in recognising interpreter needs [22] and organizational aspects [3].

### Policy changes

Use of professional interpreters in encounters with allophone patients is not always required and currently not universally feasible. Still, in general but especially in delicate, complex or important situations or in the absence of trustworthy lay interpreters, professional interpreters need to be used more frequently and therefore made accessible in a timely manner to primary care physicians.

The political environment and financial resources may influence which strategies, if any, are adopted and explain, besides the longer duration of projects in place, the seemingly better coverage of interpreter costs and lower rates of unmet interpreter needs in some French-speaking regions. Ideally, other settings will inspire themselves from successful examples such as Geneva and Vaud, giving full coverage to asylum seekers and refugees in need, or even aim for full coverage with interpreting services for primary care such as Grisons and Australia. While paying interpreters for newly arriving asylum seekers may be a message politically easier to convey, the wish for a greater public health impact may require making interpreter services available free of charge to additional groups of migrants. Assuming that physicians know best which patients benefit most from professional interpreter interventions, allocating each primary care physician a maximal amount per year for interpreter services may be a reasonable first step when universal coverage is not financially or politically reachable – though this would come with higher administrative efforts to authorities than full coverage. The need remains to ensure that existing offers for free interpreting are not reduced.

There are multiple arguments for increasing efforts aimed at appropriate interpreter use: Improved communication, intercultural understanding and quality of care [1, 11] the possibility to actually do proper health promotion, the potential of saving costs [4, 11], ethical [23] and legal aspects [24]. For preventive efforts [2] to be successful in improving overall population health, e.g. regarding chronic disease, it will be necessary to address the language barrier. This is particularly important with national and international literature pointing at healthy lifestyles [25] and higher morbidity [26, 27], lower awareness of the harmfulness of certain behaviours such as smoking [28] and poorer access to certain preventive measures in migrants [29, 30] and a substantial burden of disease already present in young migrants [31, 32]. Political efforts are therefore warranted to further improve access to interpreter services also in primary health care.

### Study limitations

There is a potential for selection bias concerning the physicians’ questionnaire as return rates were low (±11%). Physicians facing language barriers more frequently may have been more likely to respond. This may have influenced rates of unmet interpreter needs reported but is unlikely to have affected the trends between cantons. Furthermore, we mainly present findings only concerning physicians facing language barriers, so even if there was a selection bias towards physician facing language barriers, this does not pose a problem for those results. Total numbers were adequate for analysis.

Replies from interpreter agencies were few but rather homogeneous, confirming local findings. Though not representative, findings clearly indicate that for interpreter projects to be successful, offered services need to first be made available matching demand, which implies also including agencies in the planning process and potentially strengthening their resources. Poor feedback from interpreting agencies despite multiple attempts may indicate their need for support with increasing and changing demands linked to increased international migration.

Responses by cantons may have been influenced by social desirability and the fact that even within these local administrative areas knowledge on projects was not always widely available, indicating the need for better communication. Although we have not had feedback from all cantonal authorities, for some, we managed to get information indirectly. Indicated unmet interpreter needs for different cantons in the physicians’ questionnaire suggest that we have identified all important types of primary care interpreter financing currently existing in Switzerland.

Although our findings on barriers and solutions focus on Switzerland, they are most likely also relevant for other Western countries. A limited number of studies on barriers to adequate professional interpreter use exists mainly focusing on the hospital setting: factors identified - though similar - may vary in importance depending on the setting (cost, availability of interpreters etc.) [33]. Cost, for example, has also been identified as an important barrier in studies from the United States, the inconvenience of organising an interpreter also in Australia, where cost is no longer relevant, and the United States [33]. Still, key elements described in our study are likely worth considering in most settings for the implementation of policies to improve access to professional interpreters in primary care to be successful. While nationally and internationally [19, 34] efforts exist to make interpreter services easily accessible free of charge, research on the implementation of interpreter policies for primary care is extremely scares [19, 20]. Experiences with different interpreter policies made in Switzerland may therefore be of interest to policy makers in other host countries.

## Conclusion

Adequate financing of professional interpreters is the basis to addressing the language barriers in healthcare. To be successful, organizational aspects improving accessibility and availability, education of healthcare providers and comprehensive information also need to be addressed by policies aiming at increasing adequate interpreter use. Different examples of successful interventions to increase professional interpreter use exist, some focusing only on asylum seekers and refugees, some focusing on the need independent of circumstances of stay in the host country. Experiences made with interpreter policies can be used to inform improved interpreter policies.

## Supplementary information

**Additional file 1.** *Questionnaire for Physicians * Questionnaire for physicians on interpreter use, knowledge, attitude and perceived barriers as well as desired interpreter services.

**Additional file 2.** *Questions for Interpreter Agencies * Questionnaire for interpreter agencies on services provided and financial instruments known.

## Data Availability

The datasets generated and analyzed during the current study is available from the corresponding author on reasonable request.

## Abbreviations

FD: family doctor / general practitioner
NGO: non-governmental organization
PCP: primary care paediatrician
