# Supplementary material for: Barriers to and solutions for addressing insufficient professional interpreter use in primary healthcare
Source: BMC Health Serv Res. 2019 Oct 25;19:753. doi: 10.1186/s12913-019-4628-6 (PMC6815061; doi:10.1186/s12913-019-4628-6)
Supplement: Supplementary file 1 — Additional file 1. *Questionnaire for Physicians * Questionnaire for physicians on interpreter use, knowledge, attitude and perceived barriers as well as desired interpreter services. [file 12913_2019_4628_MOESM1_ESM.pdf]

English Translation of Online-Questionnaire

The online version included a skip-pattern not fully represented here, ensuring that only physicians concerned with a question would be presented with the question.

1

Please specify your function

If you work both, in a hospital and in an out-patient primary care practice (private practice), please only consider your work at the primary care practice.

□ Family doctor (FD in private practice (out-patient general primary care outside of hospitals (incl. policliniques)
□ Primary care paediatrician (PCP in private practice (out-patient general primary care outside of hospitals (incl. policliniques)
□ Internal Medicine Specialist working in a hospital without work in primary care practice
□ Paediatrician working in a hospital without work in primary care practice
□ No clinical work (Research, Retired)

When mentioning the patient, for the purpose of this questionnaire, we also include the parent/main caregiver.

When talking about professional interpreters we also included qualified mediators and intercultural interpreters.

During your medical practice you most likely also take care of patients and their families who may not be able to communicate in the local language or a common alternative language (e.g., English, other national language) or who only understand or speak it insufficiently well for a medical encounter (consultation with language barrier).

2

How often during the last year did you do consultations with a language barrier hindering direct communication of good quality with the patient or in the paediatric setting the main care giver and a school-aged child or teenager)?

Please also include cases where someone (no matter whom) did interpret, but you were not able to speak well with the patient directly, or in the paediatric setting with the main care giver who would usually provide the information

□ At least once a week
□ At least once a month (12-51x/year)
□ At least once a year (1-11x/year)
□ Less than once a year

3
Conditional Question appearing only depending on answer above
Try to quantify the number of consultations with a language barrier per month._______.

4
Conditional Question appearing only depending on answer above
Try to quantify the number of consultations with a language barrier per year: ______.

5
Conditional Question only appearing for those who face language barriers
How do you communicate in case of a language barrier?

	never	rare (< 15%)	sometimes (app. 15-50%)	often (app. 50-85%)	frequently (>85%)	
Sign language	○	○	○	○	○	○
One parent interprets for the other one. (paediatric consultations)	○	○	○	○	○	○
Adult family members /friends interpret	○	○	○	○	○	○
Minor (patient or family member) interprets	○	○	○	○	○	○
Professional Interpreter on site	○	○	○	○	○	○

	never	rare (< 15%)	sometimes (app. 15-50%)	often (app. 50-85%)	frequently (>85%)	
National Phone-interpreter Service	○	○	○	○	○	○
Patient/ Family call acquaintance who interprets using a mobile phone	○	○	○	○	○	○
Google translate, Linguee or similar program	○	○	○	○	○	○
Video Interpreter Services	○	○	○	○	○	○
Someone at private practice speaks the language	○	○	○	○	○	○
Contents oneself with poor understanding in local or alternative language (limited communication)	○	○	○	○	○	○
Visual Support, Brochures	○	○	○	○	○	○

6
Conditional Question only appearing for those who face language barriers
How often are professional interpreters used during your consultations? Try to Estimate.
If in the questionnaire we use the term professional interpreters this includes we refer to all forms of professional interpreting – on site and phone interpreting. Please, consider all interpreter interventions, independent of who initiated / organized their intervention.

□ At least once a week
□ At least once a month (12-51x/year)

□ At least once a year (1-11x/year)
□ Less than once a year

7
Conditional Question depending on reply above
Try to quantify the frequency per month (professional interpreters)

__________ per month

8
Conditional Question depending on reply above
Try to quantify the frequency per year (professional interpreters)

___________ times a year

9
Which of the following situations did occur at your practice due to language related communication difficulties at least once during the past year? (language incongruent consultation without a professional interpreter present)

□ Difficulties in determining diagnoses and therapy (difficulties taking history)
□ Additional exams ordered due to difficulties to obtain a clear patient history
□ Referral to emergency room due to insufficient level of communication
□ Hospitalization because of compliance uncertainty due to a lack of language comprehension
□ Prolonged consultations
□ Uncertainty, if all has been understood
□ Renounced giving certain explanations regarding disease and treatment plans
□ Renounced giving preventive advice
□ Uncertainty, if information is being kept back by lay interpreters (unsure if patient or parent who does not speak the language is fully informed)
□ Difficulties discussing delicate topics (e.g., violence, psychological or intimate aspects) using lay interpreters (privacy)
□ Impression of being unable to provide quality care due to the language barrier
□ Uncertainty that all has been understood

10

all:
During the last year did you face intercultural uncertainty?

Uncertainty as to how to interact with people of a different culture, uncertainty as to whether different disease concepts were understood

☐ No, not relevant
☐ Yes
☐ Don't know / no answer

Professional interpreters can improve communication, help reduce intercultural barriers and make it possible for patients or parents to communicate „directly" and well (good quality) with the physician (no information withheld, possibility to discuss personal topics)

11
How often would you ideally appreciate the help of a professional interpreter (on site or via phone interpreting) at your consultation but do not have this?
Please include on site and phone interpreting, independent of who would initiate and organize the interpreter. (unmet need)

□ At least once a week
□ At least once a month (12-51x/year)
□ At least once a year (1-11x/year)
□ Less than once a year

12
Conditional Question depending on reply above
Try to estimate the frequency per month (interpreter service desired but currently not used)

______ per month

13
Conditional Question depending on reply above
Try to estimate the frequency per year (interpreter service desired but currently not present)

______ per year

14

Conditional question

Do you ever request (initiate) the presence of an interpreter?

□ No, never
□ Only phone interpreting services
□ Only on site interpreter services
□ Yes, both

15

Conditional question

What is the main reason for not requesting (initiating/organizing) the presence of an interpreter?

□ Insufficient financial coverage
□ Organizational aspects
□ Can do without
□ Unsure where/how to organize a professional interpreter/ Service not known
□ I do not treat patients with a language barrier

16

Conditional question

In case you arranged for an interpreter, who would cover the costs?
□ Myself
□ The patient
□ Health insurance
□ Fund/non-governmental organization
□ Authorities

17

all

Are you aware of any means of cost coverage for interpreter services arranged for by private practices (financial coverage)?

Independent of if you use them or not
□ Yes
□ No
□ I don’t know

□ Yes, but I do not have access

18
Conditional question depending on answer above
Please, specify:
Who covers the cost? ____________

For which group of patients? ____________

19
Conditional question depending on answer above
Have you used the offer already?

□ Yes, satisfied
□ Yes, service needs improvement
□ No, no access
□ No, services is inconvenient
□ No, no need for this service

20
Do patients sometimes show up with externally organized professional interpreters?
(Presence of interpreter not arranged for /initiated by private practice)

□ Yes
□ No
□ I do not know

21
What percentage of interpreter assignments at your consultation are organized externally (not initiated by the private practice)?
None arranged for by the private practice, all exclusively organized externally without involvement of the private practice = 100%

22
Which category of patients sometimes show with externally organized professional interpreters?
□ Asylum-seekers

□ Dismissed Asylum seekers
□ Temporary admissions / F-permit
□ Recognized Refugees (B-permit)
□ Person in asylum process /refugee (not specified)
□ Diplomatic staff
□ Employee: Employer/Company organizes interpreter for employee
□ Other

23
Who initiates/pays for the interpreter
Please chose and explain if necessary in the free text field
□ Asylum/reception center
□ Canton/Authorities
□ NGO/Foundations

24
What are (other) relevant reasons for not using qualified interpreters more frequently?
Please also mark already mentioned reasons
□ Not used to working with interpreter
□ Not used to working with professional interpreters
□ Lack of financial coverage
□ Organization: cumbersome
□ Professional interpreters not available within requested time
□ Language not available (limited availability within reasonable time)
□ Minimal duration of interpreter service too long (would only need it for a shorter period of time)
□ Not worth the effort/ can do without
□ Language barrier not recognized or underestimated previous to consultation
□ Patients, who d have the right to be accompanied by a professional interpreter, are sent without
□ Patients usually bring a private person who translates anyway
□ Interpreting increases consultation duration
□ Limited trust in professional interpreters (information withheld, interpreter adds info, poor quality)
□ Uncertainty if patients show up (one organizes the interpreter and the patients does potentially not show)
□ Patients do not want professional interpreters
□ I do not know where/how to organize services
□ No barriers

25
For which group do you most frequently observe unmet interpreter needs , thus relevant linguistic barriers making communication with the patient (or family in the paediatric) challenging.
maximum 3 replies:

- ☐ Asylum Seekers and rejected asylum seekers
- ☐ Recognized Refugees (B-permit) and Temporary Admissions during the first years
- ☐ Asylum-seekers /Refugees without specification of status
- ☐ Undocumented migrants
- ☐ New arrivals (Work migrants, Expats, Family reunions; non asylum)
- ☐ Tourists
- ☐ Person from other Swiss language region
- ☐ First generation migrant who despite long stay does not speak well enough for a complex medical encounter
- ☐ None

26
Imagine you would receive access to a limited number of free on site interpreter sessions or free phone interpreter minutes that you can dispose of freely. Would you use them?

☐ Yes, ☐ No ☐ I don’t know /maybe

27
Would you prefer free on site interpreting or free phone interpreting minutes at your disposal?
- ☐ No preference
- ☐ On site interpreter services
- ☐ Phone interpreter services
- ☐ Preferably a mixture (both)

A few more questions at the end:

28
What canton do you work in mainly?

If multiple languages are spoken in a canton, chose the one indicating the correct language part depending on the language that you use more frequently.
□ AG, □ AI, □ AR, □ BE-Deutsch, □ BE-Français, □ BL, □ BS, □ FR-Deutsch, □ FR-Français, □ GE, □ GL, □ GR-Deutsch, □ GR-Rätoromanisch,□ JU, □ LU, □ NE, □ NW, □ OW, □ SG, □ SH, □ SO, □ SZ, □ TI, □ TG, □ UR, □ VD, □ VS-Deutsch, □ VS-Français, □ ZG, □ ZH

29
Where is your work place situated?
□ City
□ Suburbs of a city
□ Country-side

30
What approximate proportion of consultations at your private practice concern patients with a migrant background (for adults 1. generation, for paediatrics also 2. generation)?

___________%

31
At what percentage do you work in the private practice?

_____%

32
Are you a ...?
□ Man
□ Woman
□ No reply

33
We are at the end of the questionnaire: If you have any suggestions or comments regarding the use of interpreters, you may write them here (optional):

____

34
Conditional question appearing only if intercultural difficulties present
In case you still have time for a last question (otherwise click to end the questionnaire): You have mentioned having faced intercultural uncertainties, what were they?
Your Info can help improve training in intercultural medicine.

The END

The language used to fill in the questionnaire was also registered.
